# Identification and whole-genome characterization of a recombinant Enterovirus B69 isolated from a patient with Acute Flaccid Paralysis in Niger, 2015

**DOI:** 10.1038/s41598-018-20346-9

**Published:** 2018-02-01

**Authors:** Maria Dolores Fernandez-Garcia, Manasi Majumdar, Ousmane Kebe, Kader Ndiaye, Javier Martin

**Affiliations:** 1 Institut Pasteur, Dakar, Senegal; 2 Division of Virology, National Institute for Biological Standards and Control, Potters Bar, Hertfordshire, United Kingdom

## Abstract

Enterovirus B69 (EV-B69) is a rarely reported type and till date, only the full-length genome sequence of the prototype strain is available. Besides the prototype strain, only limited VP1 sequences of this virus from Africa and India are available in GenBank. In this study, we analyzed the full-length genome sequence of an EV-B69 strain recovered from a patient with acute flaccid paralysis in Niger. Compared with the EV-B69 prototype strain, it had 79.6% and 76.3% nucleotide identity in the complete genome and VP1 coding region, respectively. VP1 sequence analyses revealed also high variation in nucleotide similarity (68.9%–82.8%) with previously isolated EV-B69 strains in India and Africa. The great genetic divergence among EV-B69 strains indicates that this type is not a newly emergent virus, but has circulated for many years at low epidemic strength. Phylogenetic incongruity between structural and non-structural regions and similarity plot analyses revealed that multiple recombination events occurred during its evolution. This study expands the number of EV-B69 whole genome sequences which would help genomic comparison for future studies to understand the biological and pathogenic properties of this virus, assess its potential public health impact and comprehend the role of recombination in the evolution of enteroviruses.

## Introduction

Enteroviruses (EVs) are members of the family *Picornaviridae*, genus *Enterovirus* and are among the most common human viruses^1^. On the basis of genome organization, biological properties and phylogenetic clustering, EVs have been assigned to four species EV-A to EV-D including 116 types^2^. EV-B has the most members (63 types). EVs are small viruses with no envelope and a single positively stranded RNA genome of about 7,500 nucleotides (nt) in length and with one functional open reading frame (ORF) flanked by 5′ and 3′ untranslated regions (UTRs). The ORF encodes a viral polyprotein that is processed to give rise four structural proteins, VP1 to VP4, and non-structural proteins (2 A to 2 C and 3 A to 3D)^1^. The structural protein VP1 is the most external capsid protein which contains major viral epitopes recognized by neutralizing antibodies. EV classification relies on the high nucleotide sequence divergence within the capsid protein VP1^3^. EVs are considered to belong to a given type when they share more than 75% nt and 88% amino acid (aa) identity in the VP1 coding sequence with the prototype strain of this type. if they share less than 70% nt identity in this region then they are classified into a new type^3,4^.

EVs can cause symptoms ranging from mild febrile illness to severe and potentially fatal diseases that affect mostly infants and children such as myocarditis, encephalitis, aseptic meningitis, or poliomyelitis-like acute flaccid paralysis (AFP)^1^. One of the key strategies for the eradication of poliomyelitis is AFP surveillance^5^. Now that the Global Polio Eradication Initiative has brought poliomyelitis cases to the threshold of eradication, non-polio enteroviruses (NPEVs) are considered as one of the associated causes of AFP worldwide. In those countries without EV surveillance systems, such as those from Africa, AFP surveillance turns out to be the only source to study the circulation of NPEVs in the population. Previous studies in Africa had described the type distribution and molecular epidemiology of NPEVs using stool specimens of patients with AFP^6–9^.

The EV-B69 prototype strain was first isolated in the United States in 1959^10^ and fully sequenced by Oberste *et al*.^11^. Subsequently, other EV-B69 strains were isolated from AFP patients in Central African Republic^8^ (2002; n = 1; JN255659), Chad^9^ (2008; n = 3; JX417814-6), Cameroon^9^ (2008; n = 1; JX417813) and India^12,13^ (2008–09; n = 14; JN203993–04, JX519427–8). Six other strains were isolated in Nigeria from asymptomatic children^7^ (2003; n = 6; GQ496558–63) and two additional strains from India were isolated from encephalitis cases in 2011^14^ (KT716276–7). Identification of these strains was done via sequencing the partial VP1 region except for two Indian strains for which the sequence of the entire VP1 region is available in the GenBank database. To date, the complete genome sequence of the prototype strain Toluca-1 (AY302560) is the only one that has been reported. Here we report the identification and whole-genome characterization of an EV-B69 strain (15_491) isolated in 2015 from an AFP patient during polio surveillance activities. This is the first report of EV-B69 in Niger.

## Results

### Sample collection, viral isolation and molecular typing

The study strain EV-B69 15_491 was isolated from a stool sample from a child living in the district of Keita, province of Tahoua in Niger. The child was a 4-year-old girl with AFP. The flaccid paralysis symptoms appeared in September 10^th^, 2015, in the lower limbs with fever. Case investigation and sample collection were done in October when the patient still presented paralysis. The EV-B69 15_491 strain was isolated on RD cells after a complete EV-like cytopathic effect (CPE) was observed. It did not produce CPE in the L20B cell line. Only EV-B69, and no polioviruses or other enteroviruses were detected from the two samples collected 24 hours apart from the patient. The type was determined by sequencing the 5′ end of the VP1 capsid coding region^3,15–17^ which was analyzed by comparative nucleotide alignment with strains available in GenBank (BLAST) and by an online typing tool. The isolate 15_491 was identified as an EV-B69.

### Whole genome analysis

The nearly complete genomic sequence of EV-B69 strain 15_491, from nt 35 to nt 7392 (Toluca-1 numbering) was obtained by high-resolution analysis. Results were identical following *de novo* assembly or reference-guided assembly using a curated enterovirus sequence database (data not shown). *De novo* assembly produced a main contig generating a consensus sequence of 7,212 nucleotides in length. This contig contained 124,390 reads (83.8% of the total assembled reads) and had very low genetic variability as judged by single nucleotide polymorphism analysis (data not shown). Our results were comparable to those describe by Montmayeur *et al*.^18^ analyzing whole genomes of poliovirus grown on RD cells. The initial partial VP1 sequence described in the previous section was identical to that obtained by deep sequencing. The extreme ends of the genome were also sequenced by the Sanger method as coverage by deep sequencing was low in these areas resulting in a final sequence of 7,354 nucleotides in length. Filtered reads were re-mapped to this sequence to obtain a final contig. The sequence coverage of filtered reads across the genome is shown in Supplementary Figure S1. The consensus sequence includes a 5′UTR of 708 nt, a single ORF of 6561 nt encoding a single polyprotein of 2187 aa, and a 3′UTR of 84 nt. The overall base composition of the strain 15_491 genome was 28.1% A, 24.6% G, 23.5% C, and 23.8% U. The nt sequence of strain 15_491, prototype Toluca-1 and all prototype strains belonging to EV-B species were aligned and compared. Table 1 shows the nucleotide sequence and deduced amino acid sequence identities between them. The sequence of the complete VP1 coding region of strain 15_491 displayed 76.3% nt and 93.7% aa similarity with the EV-B69 prototype strain Toluca-1, confirming that strain 15_491 is an EV-B69 (>75% and >88% VP1 nt and aa identity respectively within a type). In contrast, it shared less than 75% (60–71.2%) nt identity and less than 88% (56.3–79.5%) aa identity in the VP1 with other EV-B types (Table 1). Comparison between strain 15_491 and prototype Toluca-1 showed that the genome of strain 15_491 was collinear with that of the prototype strain, except for two deletions at nt 96 and 126 in the 5′UTR, three deletions at nt 3304–3306 at the end of the VP1, two deletions at nt 7332–7333 in the 3′UTR and two insertions at nt 7310 and 7320 in the 3′UTR. The overall variation between the sequenced 15_491 strain and Toluca-1 at nt and aa level was 20.4% and 4.3%, respectively. Strain 15_491 shared 78.1%, 80.8% and 79.7% nt identities in the P1, P2, and P3 coding region with the EV-B69 prototype strain, respectively.

**Table 1:** Nucleotide sequence and deduced amino acid sequence identities between the study strain 15_491, the EV-B69 prototype strain Toluca-1 and other prototype strains belonging to EV-B.

	Identity with prototype Toluca-1 (%)		Identity with other EV-B (%)	
Region	**Nucleotide**	**Amino acid**	**Nucleotide**	**Amino acid**
5′UTR	82.8	—	76.2–82.6	—
VP4	77.6	94.3	67.1–80	74.3–94.3
VP2	78.8	96.1	65.5–73.6	71.2–84.5
VP3	79	95.8	62.9–70.3	69.6–84.1
VP1	76.3	93.7	60–71.2	56.3–79.5
2 A	77.8	90	73.5–82	88.6–96
2B	80.8	92.9	74–83.2	88.8–94.9
2 C	82	98.5	79.3–83.9	96.9–99
3 A	76	95.5	73–82	89.8–97.7
3B	81.8	95.4	72.7–89.4	90.9–100
3 C	79.6	96.2	76.3–85	93.9–99.4
3D	80.4	96.9	77.1–87	95.2–98.9
3′UTR	77.9	—	78.1–86.2	—

### Phylogenetic analysis

**Figure 1:**
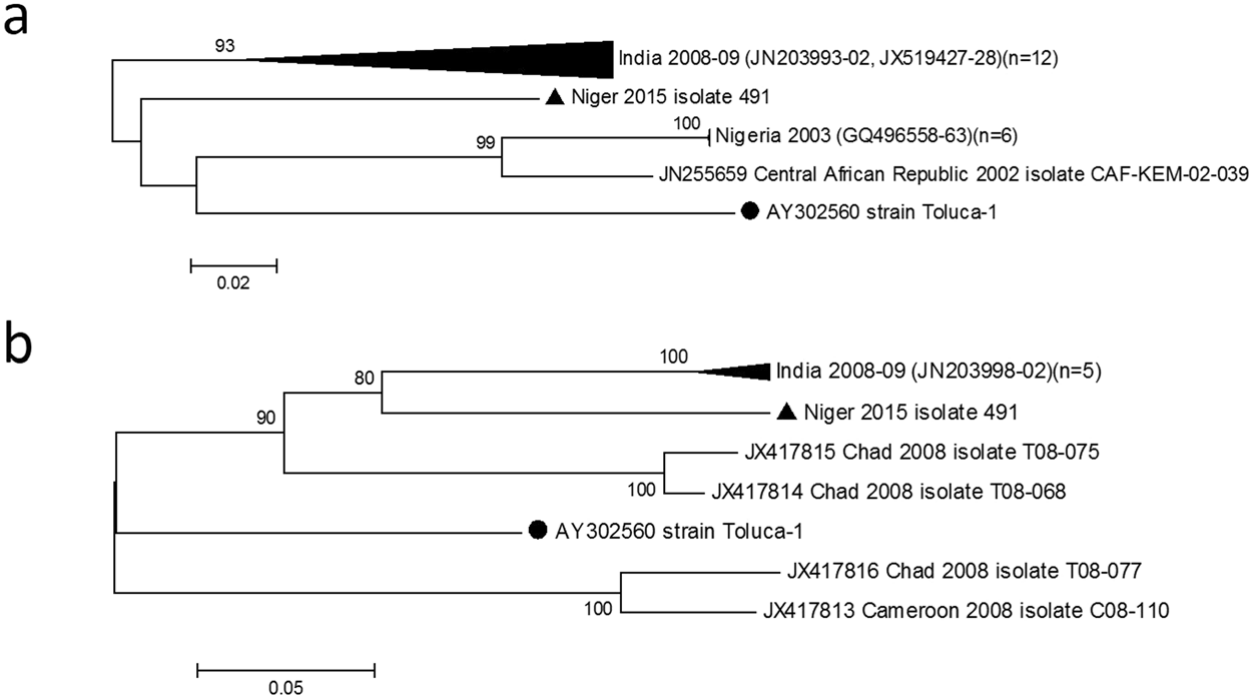
Phylogenetic tree inferred with (**a**) partial 5′ VP1 region nucleotide sequences (303 bp, nucleotide positions 2553–2857) and (**b**) partial 3′ VP1 region nucleotide sequences (328 bp, nucleotide positions 2979–3308) of global EV-B69 isolates. The black circle indicates the prototype strain; the black triangle indicates EV-B69 isolate in this study. The tree was constructed using the Neighbour-Joining method and Kimura-two parameter model. The bootstrap support values were calculated for 1000 replicates and bootstrap support >80% are indicated in nodes.

In the GenBank database there are 28 total or partial sequences of the VP1 region of EV-B69 strains available. Because there are only three complete VP1 sequences, we selected all the EV-B69 strains with more than 300-bp (n = 24) deposited in the GenBank database for phylogenetic analysis. Phylogenetic trees were generated from the 303-nt (nt position 2553–2857) partial 5′ VP1 region (Fig. 1a) and from the 328-nt (nt position 2979–3308) partial 3′ VP1 region of selected strains (Fig. 1b). In terms of nt identity, strain 15_491 was most closely related in the partial 5′ VP1 region to African strains from Central African Republic and Nigeria (mean *p*-distance of 0.193 SE ± 0.027) than to Indian strains (mean *p*-distance of 0.220 SE ± 0.027). However, in the partial 3′ VP1 region, strain 15_491 was most closely related to Indian strains (mean *p*-distance of 0.217 SE ± 0.028) than to the African strains from Chad T08–075 and T08–068 (mean *p*-distance of 0.243 SE ± 0.03). Overall, strain 15_491 has an average *p*-distance in the 5′ and 3′ VP1 region of 0.173 and 0.241 from the other EV-B69 strains, respectively, suggesting great genetic divergence among them. In terms of aa similarity, the study strain was similar to both African and Indian strains (mean *p*-distance of 0.021 SE ± 0.014 and 0.023 SE ± 0.013, respectively) in the 5′ VP1 region whereas it was more closely related to Indian strains (mean *p*-distance of 0.124 SE ± 0.031) in the 3′ VP1 region than to the African strains from Chad T08–075 and T08–068 (mean *p*-distance of 0.190 SE ± 0.04).

**Figure 2:**
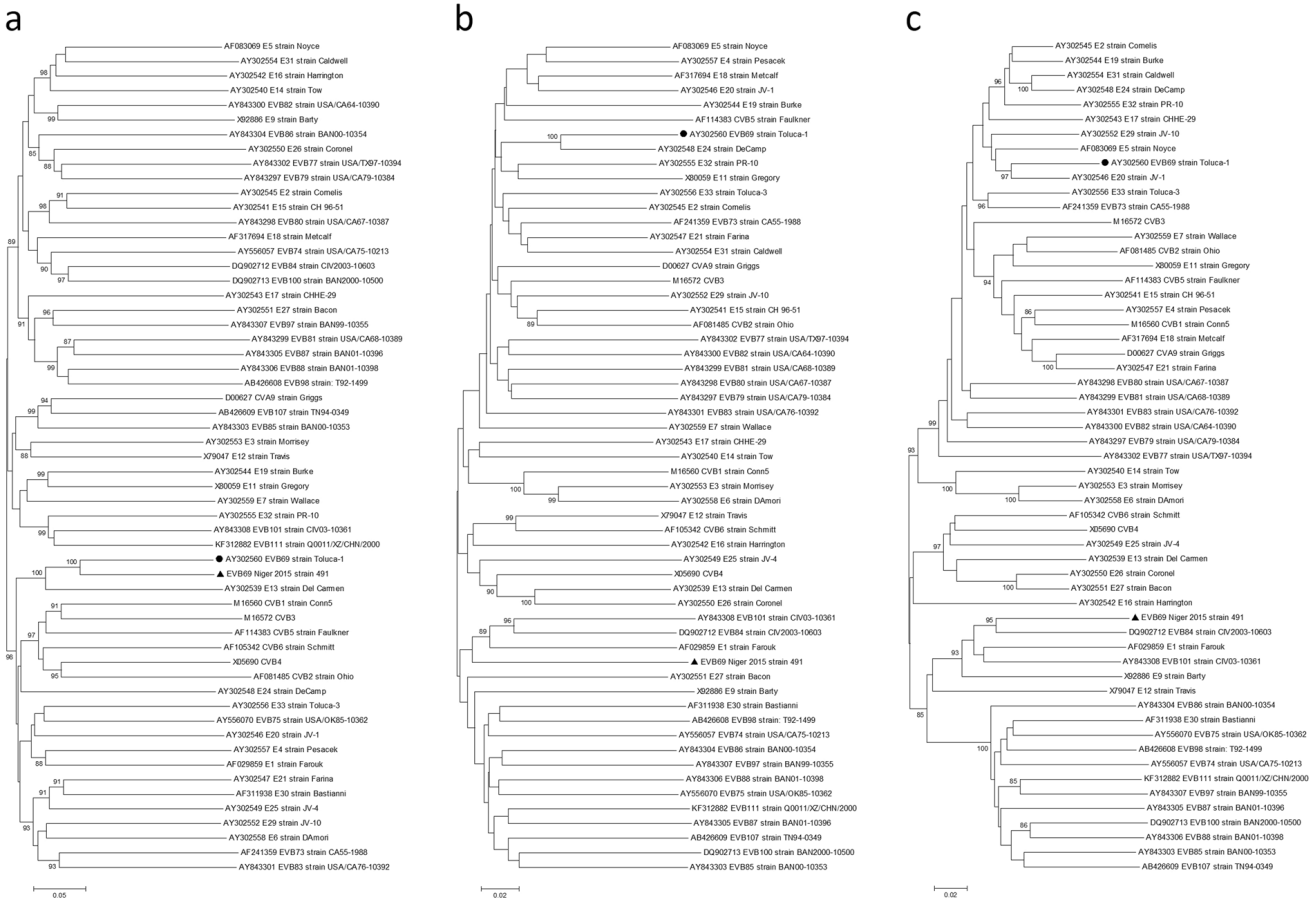
Phylogenetic relationships of the EV-B69 study strain 15_491 and other EV-B prototype strains. The phylogenetic trees were performed from the nucleotide sequence alignment of a ~2560, 1733 and 2268 bp-sized nucleotide sequence data within the P1 (**a**), P2 (**b**), and P3 (**c**) coding regions, respectively, by using the neighbor-joining algorithm of the MEGA 5.0. software. The reliability of tree topology was estimated by using 1000 bootstrap replicates. Bootstrap values are indicated if higher than 80%. Scale bar represents nucleotide substitutions per site. Black triangles indicate the strains from this study. Black circle indicates prototype strain. GenBank accession numbers for published sequences are shown in the tree.

Phylogenetic analysis based on the P1, P2 and P3 coding regions of strain 15_491, EV-B69 prototype Toluca-1 strain and all EV-B prototype strains available in GenBank database was conducted to investigate their genetic relationships (Fig. 2). As expected for the P1 capsid coding region, strain 15_491 clustered with the prototype strain in a monophyletic group with a bootstrap value of 100%, confirming the preliminary molecular typing results (Fig. 2a). However, for the nonstructural P2 and P3 coding regions, strain 15_491 did not cluster with the prototype Toluca-1 but with other EV-B types. Highest similarity was shared with prototype strains of EV-B101, EV-B84 and E1. These incongruent tree topologies between the structural and nonstructural regions suggested that recombination between EV-B69 and other EV-B types might have occurred.

### Recombination analysis

**Figure 3:**
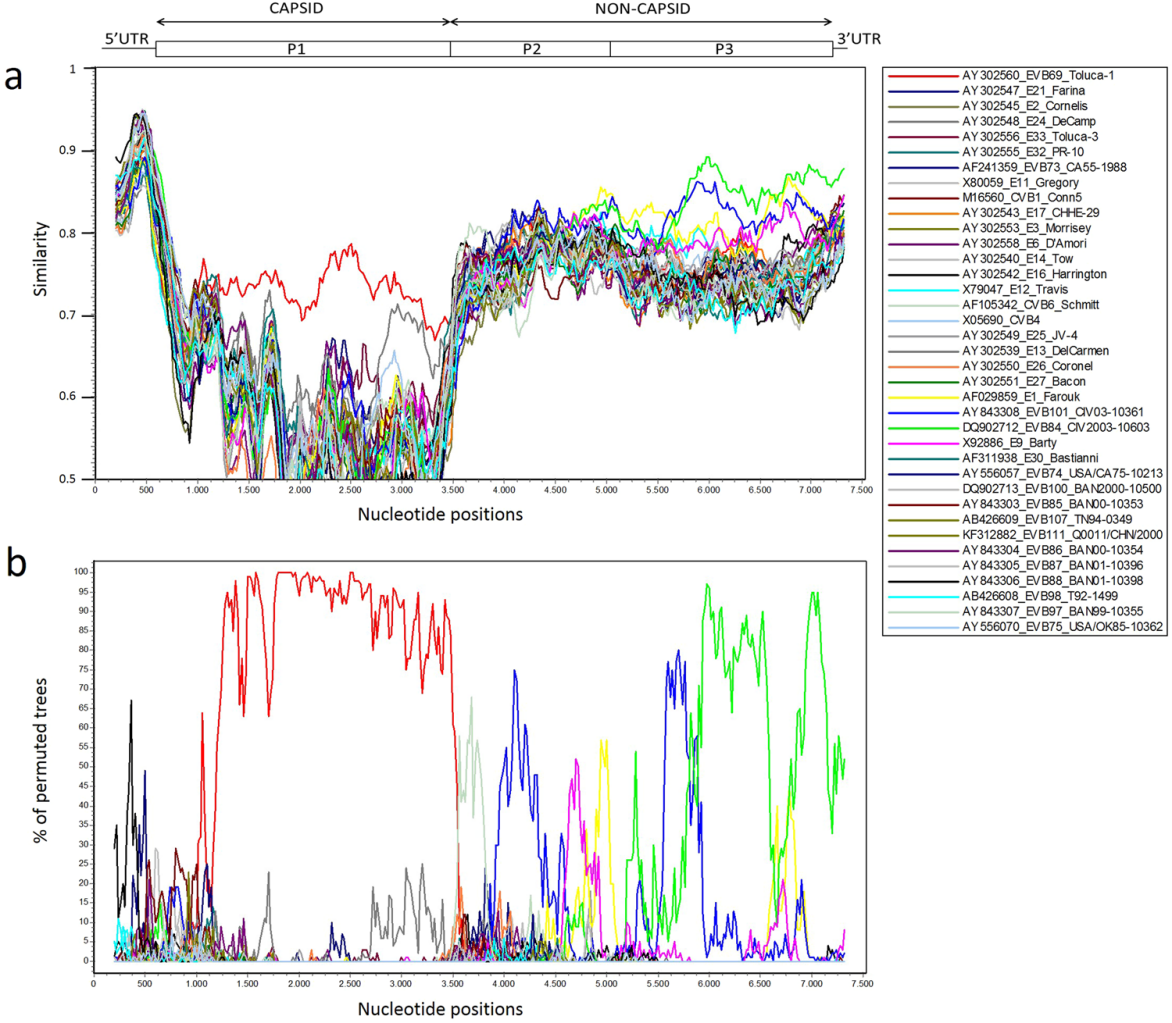
Plot of similarity (**a**) and bootscanning analysis (**b**) of the EV-B69 study strain 15_491 with EV-B prototype strains. Approximate nt positions in the enterovirus genome are indicated. The enterovirus genetic map is shown in the top panel. The analyses were calculated by SimPlot version 3.5.1 using Kimura distance method in a sliding window of 400 bp moving in steps of 20 nucleotides. The genome of study strain 15_491 serves as a query sequence.

**Figure 4:**
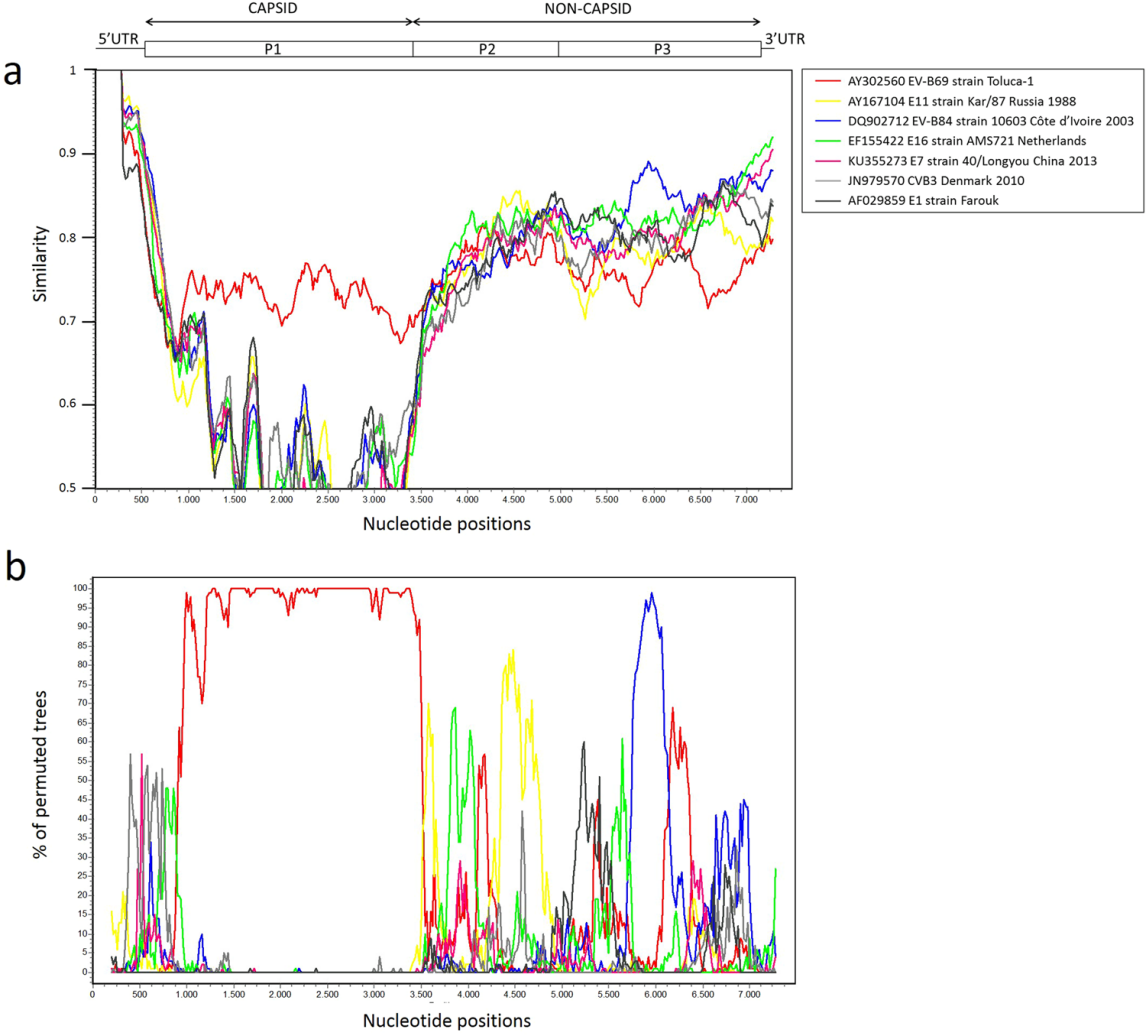
Plot of similarity (**a**) and bootscanning analysis (**b**) of the EV-B69 study strain 15_491 with closely related strains. Approximate nt positions in the enterovirus genome are indicated. The enterovirus genetic map is shown in the top panel. The analyses were calculated by SimPlot version 3.5.1 using Kimura distance method in a sliding window of 400 bp moving in steps of 20 nucleotides. The genome of study strain 15_491 serves as a query sequence.

To confirm the existence of recombination events, similarity plot and bootscanning analysis were conducted against other EV-B prototype strains (Fig. 3). As expected, strain 15_491 had the highest similarity with the EV-B69 prototype strain in the P1 capsid coding region. However, within the nonstructural P2 and P3 regions, comparison revealed that strain 15_491 had the highest similarities with EV-B84 (89%), EV-B101 (86.1%) and E1 (86.7%). Bootscanning analysis confirmed the existence of multiple recombination events between the Niger 15_491 strain and related EV-B types. Because of the large number of EV-B strains in GenBank, closely related types were selected using BLAST online. The non-structural region of the study strain 15_491 was used for BLAST, and the sequences with the highest similarities and with complete genomes were used in the recombination analysis (Fig. 4). Resulting strains were an E11 from Russia (AY167104), an EV-B84 from Côte d’Ivoire (DQ902712), an E7 from China (KU355273), an E16 from the Netherlands (EF155422) a CVB3 from Denmark (JN979570) and the prototype E1 strain. The similarity plot analysis revealed possible multiple recombination events further confirmed by bootscanning analysis and a relatively higher percent support value (>90%) at the 3′-end of P3 and the 3′UTR for E16 and E7, respectively.

## Discussion

Retrospective investigation of stools samples from AFP surveillance is routinely carried out in the WHO Polio Reference Laboratory in Dakar, Senegal, to understand the frequency, circulation, genetic diversity, and evolution of NPEVs in West Africa^6^. The investigation comprises the isolation of NPEV strains and their molecular typing based on VP1 sequencing. In 2015, during this routine characterization of NPEVs, we isolated a rarely reported type, EV-B69, recovered from a patient with AFP in Niger, which, to the best of our knowledge, is the first report of EV-B69 in this country. Retrospective investigations of the NPEVs from AFP surveillance conducted worldwide in support of global polio eradication has allowed the identification of EV-B69 strains from Africa (Nigeria, Chad, Cameroon and Central African Republic)^8,9,19^ and Asia (India)^12,13^, suggesting a global distribution of EV-B69. However, in all these studies, EV-B69 was isolated in extremely low isolation rates. Moreover, in a previous study that analyzed stool samples from children with AFP collected through routine poliomyelitis surveillance activities between 2013 and 2014, no EV-B69 strain was identified neither in Niger nor in other West African countries^6^. In the same way in Asia, other large studies describing the epidemiology of NPEVs during large surveillance periods, have not detected EV-B69 strains^20–22^. These extremely low isolation rates worldwide suggest that EV-B69 till now is not an established pathogen neither in Africa nor in Asia, although silent transmission can be suggested. Therefore, further surveillance data on this virus in human populations, such as sero-epidemiological studies, might provide valuable information about the prevalence of this virus.

We describe here the whole-genome characterization of the Niger EV-B69 isolate. To date, there is only one complete genome sequence of EV-B69 (the prototype strain isolated in 1959) available in the GenBank database. In addition to that, a limited number of EV-B69 partial nucleotide sequences (majority VP1 coding region) are also archived. The genomic analyses of the VP1 coding region of the 15_491 EV-B69 strain revealed a significant nucleotide divergence (23.7%) from the prototype strain and >28% nucleotide differences from other EV-B published sequences. Pairwise comparison with VP1 sequences of other EV-B69 strains (African and Indian strains) also revealed a great level of genetic diversity between strains suggesting that this virus has not emerged recently and has been circulating independently for a long time.

Although, study strain 15_491 and the majority of EV-B69 strains in GenBank were isolated from AFP or encephalitis cases, it is not possible to establish a definitive causal link between the clinical symptoms and the presence of EV-B69 strain in stool samples, as NPEVs are excreted in stools for a long period of time. In this study, stools were collected 30 days after onset of paralysis and we could not collect any supplementary epidemiological information, such as residual paralysis at day 60, to conclude that EV-B69 was the causative agent. However, all virus isolation procedures were performed following WHO accredited methods and no other enterovirus isolates were revealed by our cell culture and deep sequencing analysis.

Due to the worldwide limited number of EV-B69 isolates, it is currently difficult to study its biological and pathogenic properties and its possible association with neurological diseases. More studies need to be carried out on large numbers of samples from a broader spectrum of diseases other than AFP, with complete clinical records, to study the pathogenetic determinants of this virus. Our complete genome could provide a resource for these studies.

EVs evolve rapidly (around 1–2% per nt site per year) accumulating nt substitutions, deletions or insertions which conforms EV genome diversity^23^. Of notice, strain 15_491 has three nt deletions at the end of the VP1 on comparison with the prototype strain. Because the VP1 region is exposed on the viral external surface and is involved in receptor binding and viral antigenicity, investigations on the effect of these deletions on antigenicity or pathogenicity may be needed. Recombination is also a well-known mechanism for EV evolution^11,24,25^. When different EVs infect and replicate in the same cell, recombination between parts of the genome of different types may occur resulting in viruses with new genomic combinations that may become more virulent or more transmissible. This recombination process allows EVs to create and maintain their genetic diversity and fitness^25^. In our study, full genome sequencing revealed that recombination had taken place within the non-structural coding region when compared to the prototype strain. First, the phylogenetic incongruity between the structural and non-structural regions suggested that multiple recombination events have taken place during EV-B69 evolution. Then, similarity plot and boot scanning analysis with EV-B prototype strains suggested high (>85%) percent support values for EV-B84, E1 and EV-B101. However, although our analyses suggest that the donor of the recombinant noncapsid sequence could be derived from one of these viruses, the parental strain of the noncapsid sequence could not be identified. Since the recombination frequency of EVs is very high and many prototype strains have been isolated several decades ago, more accurate predictions to identify the exact recombination partners can be obtained through analysis of closely related sequences. For this aim, we screened strains with high similarity with EV-B69 strain 15_491 in the P2 and P3 regions using the BLAST tool. Recombination events with higher (>90%) percent support values for an E16 from the Netherlands and an E7 from China were observed in the P3 and 3UTR regions. However, the similarity in the rest of the non-structural region was still not high enough to conclude that any of these viruses are the possible recombination partner. The fact that the countries where these viruses have been isolated are geographically far from Niger suggest that long-term transmission of EV-B69 has taken place, providing the spatial and temporal circumstances for recombination to occur. Lack of complete genome sequences for many EV types limits interpretation of results for recombination analysis. Therefore, sequencing of more complete genome sequences of human EV B strains worldwide will provide a clearer picture of the role of recombination in these species. Further exploration of environmental or clinical samples using deep sequencing technology would be of interest to study co-circulating EVs and their evolution by recombination.

In conclusion, this is the first report of a full-length genome of EV-B69 in addition to the prototype strain. This study expands the number of EV-B69 genome sequences in the GenBank database, which would help genomic comparison for future studies to understand the biological and pathogenic properties of this virus, to elucidate its evolutionary history and to assess its potential public health impact.

## Materials and Methods

### Ethics statement

The study used cell culture isolates of viruses recovered from stool specimens of AFP cases collected through routine poliomyelitis surveillance activities at the instigation of Word Health Organization (WHO) for public health purposes. All technical and ethical aspects were approved by WHO and the Ministry of Health of Niger. The protocol and oral consent were determined as routine surveillance activity by the steering committee of WHO in compliance with all applicable National regulations governing the protection of human subjects. Informed consent for the use of clinical samples was obtained from the legal guardians. The methods were carried out in accordance with the principles of the Declaration of Helsinki.

### Sample collection and virus isolation

The EV-B69 strain 15_491 was isolated from a stool sample collected in 2015 during the course of AFP surveillance activities in support of the global polio eradication initiative according to WHO guidelines^26^. Briefly, all AFP cases under 15 years of age should be reported immediately and investigated within 48 hours, and two stool specimens should be collected between 24–48 hours apart and within one month of the onset of paralysis^26^. The two stool samples collected with 24 h interval in October 9^th^ and 10^th^ were submitted to the WHO Reference Intercountry Laboratory for poliomyelitis surveillance in the Pasteur Institute of Dakar (Senegal) and processed following WHO standard procedures^27^. Human rhabdosarcoma (RD) and mouse L cells expressing the human poliovirus cellular receptor CD155 (L20B) cell lines were used for virus isolation. Chloroform-treated stool solution was added to each of these cell lines in culture tubes. Only viral cultures producing cytopathic effect (CPE) in RD cells and not in L20B cells were considered to contain NPEVs. When CPE was obtained, infected cells were harvested and kept frozen (−20 °C) until typing. The virus grew only in RD cells. The isolate was further analyzed for typing and full-length sequencing.

### Molecular typing

The viral RNA was extracted from the infected cell-culture using the QIAamp viral RNA mini kit (Qiagen, Germany) according to the manufacturer’s recommendations. RNA was then reverse transcribed into cDNA and tested using a semi nested reverse-transcription-polymerase chain reaction (RT-snPCR) as previously described^27^. Primer pairs AMTH-GDCL^15^ and 222–224^17^ were used for amplifying the VP1 coding region as previously described^6^. PCR product was subjected to nucleotide sequencing at Genewiz (Essex, United Kingdom) by the Sanger method using the same forward and reverse primers (224 and 222, respectively) that we used for nested PCR. The VP1 sequences obtained were compared with homologous sequences available in the GenBank database using the program BLAST (Basic Local Alignment Search Tool) in NCBI. The sample was assigned the type with the highest identity score. Type was confirmed with the RIVM genotyping tool (http://www.rivm.nl/mpf/enterovirus/typingtool/).

### Full-length genome amplification

The nearly complete genome of EV-B69 strain 15_491 was sequenced using a combination of Sanger and deep sequencing methods. Sequence-independent single-primer amplification (SISPA) of purified RNAs from infected cells and deep sequencing analysis were performed as described before^18,28–30^. RT-PCR templates were generated in a random RT-PCR reaction using primers RA01-N8 (5′- GCC GGA GCT CTG CAG ATA TCN NNN NNN N -3) and RA01 (5′- GCC GGA GCT CTG CAG ATA TC-3′). Sequencing libraries were prepared using Nextera XT reagents and sequenced on a MiSeq using a 2 × 301 paired-end v3 Flow Cell and manufacturer’s protocols (Illumina, California, USA). Raw sequence data were imported into Geneious R10 software (Biomatters, Auckland, New Zealand) and paired end reads combined. Data were filtered using a custom workflow with the following parameters: PCR primers and Nextera adaptor/index sequences were trimmed from 5′ and 3′ ends with a minimum 5 bp overlap; reads were trimmed to have an average error rate <1%, no bases with a quality of <Q30 and no ambiguities. Reads <50 nt in length were discarded; duplicate reads were removed using the program Dedup (within Geneious) and host contaminating human genome sequences were filtered out using the hg38 database^31^. Filtered reads were assembled *de novo* using stringent assembly conditions: minimum 50 base overlap, minimum overlap identity of 98%, maximum 2% mismatches per read, allowing up to 15% gaps and both pair reads mapping. The extreme ends of the genome were also sequenced by the Sanger method as coverage by deep sequencing was low in these areas. Primers:

5′end: EVB69-D25-34F CAGGAAACAGCTATGACCTTAAAACAGCYYKKGGGTTGYWCCCRCYCACAG and EBV69-D25-942R TGTAAAACGACGGCCAGTGATCTTGTGTGAAGTCCTGTCTATTTGCC 3′end: EVB69-D25-6988F CAGGAAACAGCTATGACCGATCGCTTCCTACCCGTGGCCC and NPEV-7440R TTTTTTTTTTTTCCGCACCGAATRCGGAGAA were used to generate PCR products spanning the 5′-end and 3′-end of the EV-B69 genome, respectively. The resulting contig (7354 nucleotides long) was analysed by BLAST and identified as an EV-B69 virus. As a result, we obtained the nearly whole-genome nucleotide sequence of an EV-B69 strain (from nucleotide 35 to 7392 (Toluca-1 numbering)).

### Phylogenetic analysis

The full-length sequence was aligned to the whole-genome sequence of prototype Toluca-1 and partial sequences of 28 isolates representing EV-B69 strains from Nigeria, Chad, India, Cameroon and CAR identified by search in GenBank. Alignment of sequences was performed using the BioEdit software (version 7.0.9.0) and the ClustalW multiple alignment program. The alignments (nucleotide [nt] and translated amino acid [aa] sequences) were analyzed by using BioEdit to identify specific mutations. The phylogenetic trees were constructed with the Molecular Evolutionary Genetics Analysis (MEGA5) software, version 5.0 (http://megasoftware.net/) using the Neighbor Joining algorithm with a Kimura 2-parameter model after excluding positions containing gaps and missing data from the alignments. Bootstrapping was performed with 1000 replicates. Bootstrap values of >80% were used to indicate robust support for the tree topology. Sequence divergence was determined by using MEGA5 to calculate mean pairwise distances within groups.

### Recombination analysis

The full-length genome sequence of isolate 15_491 was aligned against complete genomes of closely related strains of Human Enterovirus B Species. Similarity plot and boot scanning analysis for potential recombination were performed using SimPlot, version 3.5.1. For similarity plot and boot scanning analyses, a 400-nt window moved in 20-nt increments using a Kimura two-parameter method with a transition-transversion ratio of 2 with 1000 resampling.

### Accession codes

The sequence read archives and contig assembly are available at NCBI under BioProject Accession No. PRJNA422141. The nucleotide sequence of the complete genome of the EV-B69 strain 15_491 has been deposited in the GenBank database (Accession No. MF521674).

## Electronic supplementary material

Supplementary Figure 1
