# Supplementary material for: Identification and whole-genome characterization of a recombinant Enterovirus B69 isolated from a patient with Acute Flaccid Paralysis in Niger, 2015
Source: Sci Rep. 2018 Feb 1;8:2181. doi: 10.1038/s41598-018-20346-9 (PMC5795009; doi:10.1038/s41598-018-20346-9)

Identification and whole-genome characterization of a recombinant Enterovirus B69 isolated from a patient with Acute Flaccid Paralysis in Niger, 2015

Maria D. Fernandez-Garcia[1*], Manasi Majumdar[2], Ousmane Kebe[1], Kader Ndiaye[1], Javier Martin[2]

* Corresponding author dolores.fernandez@yahoo.es

Supplementary information

Supplementary Figure 1. Genome coverage of EV-B69 strain 15_491 genome by deep sequencing. Filtered reads were mapped to the final consensus sequence of EV-B69 strain 15_491 genome generated by *de novo* assembly and Sanger sequencing. The number of sequence reads at each nucleotide position is shown. The location of viral genes is indicated.

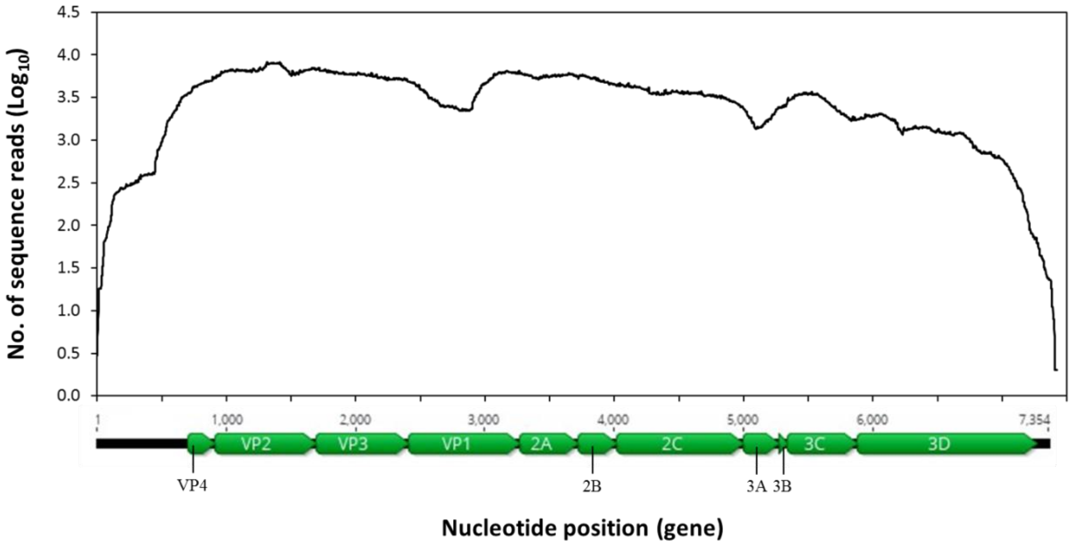

Supplement: Supplementary file 1 — Supplementary Figure 1 [file 41598_2018_20346_MOESM1_ESM.pdf]
